# Epidemiological features and spatial-temporal clustering of visceral leishmaniasis in mainland China from 2019 to 2021

**DOI:** 10.3389/fmicb.2022.959901

**Published:** 2022-08-29

**Authors:** Yuanyuan Li, Zhuowei Luo, Yuwan Hao, Yi Zhang, Limin Yang, Zhongqiu Li, Zhengbin Zhou, Shizhu Li

**Affiliations:** ^1^National Institute of Parasitic Diseases, Chinese Center for Disease Control and Prevention (Chinese Center for Tropical Diseases Research), NHC Key Laboratory of Parasite and Vector Biology, WHO Collaborating Centre for Tropical Diseases, National Center for International Research on Tropical Diseases, Shanghai, China; ^2^School of Global Health, Chinese Center for Tropical Diseases Research, Shanghai Jiao Tong University School of Medicine, Shanghai, China

**Keywords:** visceral leishmaniasis, epidemiology, China, spatial-temporal clustering, hotspot

## Abstract

**Background:**

Visceral leishmaniasis (VL) is a serious vector-borne disease in central and western China. In recent years, the number of VL cases increased gradually, particularly the mountain-type zoonotic visceral leishmaniasis (MT-ZVL). This study clarified the epidemiological features and spatial-temporal clustering of VL in China between 2019 and 2021, identified the risk areas for VL transmission, and provided scientific evidence for the prevention and control of VL.

**Materials and methods:**

The information on VL cases in 2019–2021 was collected from the Infectious Disease Reporting Information Management System of the Chinese Center for Disease Control and Prevention. The epidemiological characteristics of VL cases were analyzed. The global *Moran’s I* and Getis-ORD Gi* statistical data were processed for spatial autocorrelation and hotspot analysis in ESRI ArcGIS software. Also, spatial-temporal clustering analysis was conducted with the retrospective space–time permutation scan statistics.

**Results:**

A total of 608 VL cases were reported from 2019 to 2021, with 158, 213, and 237 cases reported each year, respectively. Of the 608 cases, there were 10 cases of anthroponotic visceral leishmaniasis (AVL), 20 cases of desert-type zoonotic visceral leishmaniasis (DT-ZVL), and 578 cases of MT-ZVL. The age of VL cases was mainly distributed in the group of subjects aged ≥ 15 years. Peasants and infants were the dominant high-risk population. The incidence peak season of VL occurred between March and May. The cases were mainly distributed in Shanxi (299 cases), Shaanxi (118 cases), and Gansu (106 cases) Provinces, accounting for 86.02% (523/608) of the total reported cases in China. Spatial analysis revealed that clustering of infection is mainly located in eastern Shanxi Province and Shaanxi–Shanxi border areas, as well as southern Gansu and northern Sichuan Province. In addition, new reemergence hotspots in Shanxi, Henan, and Hebei Provinces have been detected since 2020. Spatio-temporal clustering analysis revealed an increase in the degree of infection aggregation in eastern Shanxi Province and Shaanxi–Shanxi border areas.

**Conclusion:**

The AVL and DT-ZVL were endemic at a lower level in western China, whereas MT-ZVL rebounded rapidly and showed a resurgence in historically endemic counties. The spatial-temporal clustering analysis displayed that the high-incidence areas of VL have shifted to central China, particularly in Shanxi and Shaanxi Provinces. Integrated mitigation strategies targeting high-risk populations are needed to control VL transmission in high-risk areas.

## Introduction

Visceral leishmaniasis (VL), also known as Kala-azar, is a serious infectious disease caused by Leishmania spp. that is transmitted by the bite of phlebotomine sand flies (Alvar et al., 2006; Bates, 2007; World Health Organization [WHO], 2010), the hosts of which include animals like canids, rodents, marsupials, hyraxes, and human beings (Akhoundi et al., 2016), because VL can be fatal if left untreated in over 95% of the cases (World Health Organization [WHO], 2022). In many countries, more male than female cases are reported. The incubation period ranges from 10 days to over 1 year, and the onset of the disease is usually gradual (World Health Organization [WHO], 2010). The main clinical manifestations of this disease are usually chronic irregular fever, weight loss, hepatosplenomegaly,anemia,leukopenia, and increased serum globulin levels (World Health Organization [WHO], 2010). In 2018, a total of 83 countries or territories were considered endemic for or had previously reported cases of VL (World Health Organization [WHO], 2020), mainly distributed in South Asia, East Africa, South America, the Mediterranean Region, and Central Asia (Killick-Kendrick, 1990). VL is considered as the second most deadly parasitic disease following malaria, with 200,000–400,000 cases reported and approximately 60,000 fatalities annually (Alvar et al., 2012). In 2021, World Health Organization (WHO) listed VL as a public health problem that must be eliminated (Casulli, 2021).

In the early 1950s, VL was one of the five most serious parasitic diseases endangering human health in the People’s Republic of China. It was prevalent in 16 provinces (autonomous regions and municipalities) located north of the Yangtze River, with about 530,000 patients (Wang and Wu, 1956; He et al., 1959). In 1956, the Chinese government put the elimination of VL within a specified time limit as the top priority of public health work. Through active efforts by the government, VL was brought under control by 1960 in most areas of the country (Wang et al., 2000). In the 1980s, only a few sporadic cases remained in southern Xinjiang, Gansu, Sichuan, Shaanxi, and Shanxi Provinces (Guan et al., 2000). Since the 21st century, the epidemic situation of VL has changed with the evolvement of natural ecology and the social environment. A large number of historically endemic counties have experienced the resurgence of VL, leading to the gradual expansion of endemic scope (Han et al., 2019; Zhou et al., 2020c). During the historical period, three epidemiological types of VL, namely, AVL, DT-ZVL, and MT-ZVL, were identified in mainland China (Xiong et al., 1976; Guan and Shen, 1991).

The AVL, whose transmission cycle is from human to human, is caused by *Leishmania donovani* (Guan and Wu, 2014). This type was once seriously endemic in the plain areas of eastern and central China, including the south-eastern suburbs of Beijing, southern Hebei, Shandong, eastern Henan, northern Jiangsu, northern Anhui, Shaanxi plains, the northern Jianghan plain of Hubei, and the ancient oasis area of southern Xinjiang, where the majority of the cases were juveniles (Zhou et al., 2020d). Because the patients were the primary reservoir hosts of AVL, concurrent or consecutive cases often occurred in one household with spatial clusters, sometimes leading to outbreaks (Zhou et al., 2020d). After the control program was established, the disease was remarkably controlled in the above-mentioned regions, and is currently distributed in the oases of the plains of Kashi Prefecture, Xinjiang (Zheng et al., 2020).

The MT-ZVL, caused by *Leishmania infantum*, is currently distributed in the northern suburbs of Beijing, northern Hebei, western Henan, Shanxi, northern and southern Shaanxi, southern Gansu, and northwestern Sichuan, where a sub-type of *Ph. Chinensis* (a semiwild species) was the vector and dogs were the reservoirs with high infection rates. MT-ZVL was hypoendemic in humans and affected mainly infants under 5 years of age. The incidence rate in humans is much lower than the rate in canines, and no obvious correlation was found between the cases (Guan and Shen, 1991).

The most prominent epidemic area of DT-ZVL is located in the desert regions of southern Xinjiang, western Inner Mongolia, and northern Gansu Province, where Phlebotomus Wui (an exophilic species) was the primary vector (Guan, 1991). The incidence of the infection was sporadic and affected mainly infants under 2 years of age (Han et al., 2019). The DT-ZVL region was considered to be a natural nidus of VL, with wild animals presumed to be the source of infection (Wang et al., 2010). It had been an uncultivated desert for a considerable period of time before it was populated by immigrants who introduced agriculture cultivation and other activities. Consequently, autochthonous infantile VL occurs after immigration.

These three types of VL exhibit substantial differences in epidemiological characteristics, i.e., geographical and landscape features, ecosystem, vector species, and infectious sources (Guan et al., 2016), and therefore require targeted and timely control measures to efficiently allocate the relatively scarce resources.

In recent years, a rapid reemergence of VL has been seen in counties that had previously achieved elimination in China. It presented a significant challenge for the control of the disease. From the year 2019, public health agencies have been recruited by the government to perform epidemiological investigations on each VL case, to trace it back to their original infection place. This detailed information will facilitate more accurate risk identification than before, and analysis of epidemiological features and spatial-temporal clustering of VL.

Scholars have carried out some studies on the spatial-temporal clustering of VL. Zheng et al. (2020) executed a spatio-temporal analysis of VL in northwest China from 2004 to 2018, showing that VL is still seriously prevalent in Kashi Prefecture, Xinjiang Uygur Autonomous Region. Hao et al. (2021) conducted a spatio-temporal clustering analysis of MT-ZVL in central China. Guan et al. (2021) detected the shifting of high-incidence areas of VL through spatial-temporal distribution analysis in mainland China. However, these studies were either limited to the spatio-temporal analysis in localized provinces, or to a single epidemic type of VL, failing to elucidate the whole epidemiological features and spatial-temporal clustering of all three kinds of VL in China, so they are not conducive to guiding the prevention and control of the disease.

In this study, large-scale epidemiological investigations on each case were conducted for the first time to determine their specific infected counties. We classified VL cases into MT-ZVL, AVL, and DT-ZVL, and elucidated their epidemiological features and spatial-temporal clustering of all three different epidemic types of VL, which will be helpful for guiding the accurate prevention and control of VL.

## Materials and methods

### Data sources

In 2004, the web-based National Diseases Reporting Information System (NDRIS) was established in China. It is a network covering all the medical and health institutions at or above the township level. According to the Law of the Infectious Disease Control and Prevention, each medical and health institution in the country has to notify the cases of VL online within 24 h after the clinical cases are confirmed with the diagnostic criteria of VL in China (WS258-2006). Then the county CDC, where the cases are reported, will take responsibility to conduct the epidemiological investigation of individual cases in 7 days to determine whether the patient is an autochthonic case or not, and subsequently take countermeasures onsite according to relevant guidance. The information of the cases reported through NDRIS includes age, gender, occupation, current residential address, symptom onset date, diagnosis date, and therapeutic outcome, based on which demographic distribution, geographic distribution, temporal distribution, and the lag time between symptom onset and diagnosis can be analyzed.

In this study, VL case data, reported in mainland China from 2019 to 2021, were obtained from the National Notifiable Communicable Disease Reporting System (Zhang et al., 2017). Suspected cases of VL were excluded, and the revised final audit data of the cases were taken as the statistical data. The county with each case of contracted VL was selected as the sampling point for latitude and longitude coordinates.

### Analysis of changes in epidemic trend of visceral leishmaniasis

All VL-related epidemiological data from 2019 to 2021 were integrated into Microsoft Excel 2016, to analyze the demographic, temporal, and geographic distribution characteristics of VL cases through descriptive epidemiological methods. Joinpoint Regression (JPR) used mathematical statistical analysis to search for turning points and divided the trend of disease incidence over a long period into several statistically significant sections for analysis. In this study, the number of VL cases in 2015–2018 was obtained from the National Notifiable Communicable Disease Reporting System (Zhou et al., 2020a). We analyzed the trends in crude rates per 100,000 people of three epidemic types of VL using the JPR Program (version 4.3.1). A *t*-test was used to judge the significant difference in the incidence trend of VL during a certain period (Weir et al., 2016). The long-term trend of the linear segment is depicted in accordance with the best-fit result, and the annual percentage change (APC) values were calculated (Hou and Huo, 2013). The “crude rate” means crude incidence rate which refers to the frequency of new cases of a disease in a specific range of people over a period of time. It is a measurement that reflects the impact of disease on the health of the population and describes the disease distribution status.

### Spatial autocorrelation analysis

Spatial autocorrelation is defined as the correlation between values of a single variable at different geographical locations using a measurement of spatial clustering based on feature locations and attribute values (Kirby et al., 2017), and the closer the things or phenomena are in spatial locations, the more similar they are. ArcGIS software is mainly used to analyze the spatial clustering of diseases. In ESRI ArcGIS software (version 10.3), global Moran’s I and Getis-ORD Gi* statistical data were used for spatial autocorrelation and hotspot analysis, respectively (Bhunia et al., 2013). The Global Moran’s I statistic estimates the overall degree of spatial correlation for a dataset and is calculated using the following formula (Lun et al., 2015; Engels and Zhou, 2020).


$$I=\frac{n\,\sum_{i=1}^{n}\sum_{j=1}^{n}w_{i\,j}\,\left(x_{i}-\bar{x}\right)\,\left(x_{j}-\bar{x}\right)}{\sum_{i=1}^{n}\sum_{j=1}^{n}w_{i\,j}\,\sum_{j=1}^{n}{\left(x_{i}-\bar{x}\right)}^{2}}$$

where *I* is indicative of the Moran’s I statistic, with values ranging from -1 (perfect dispersion) to 1 (perfect correlation). Negative values indicate dispersion, positive values mean clustering, and a value of zero indicates no spatial correlation (Blazquez et al., 2018).

The Getis-ord Gi* statistic is a spatial autocorrelation index, based on a weighted distance matrix, and it determines spatial clustering of locations using high (hot spot) or low values (cold spot) with statistical significance judged by *Z*-scores and *P*-values (World Health Organization [WHO], 2010; Alvar et al., 2012; Abdullah et al., 2017). It is calculated using the following formula:


$$G_{i}^{*}=\frac{\sum_{j=1}^{n}w_{i\,j}\,x_{j}-\overset{-}{x}\sum_{j=1}^{n}w_{i\,j}}{\sqrt[{s}]{\frac{\left[n\,\sum_{j=1}^{n}w_{i\,j}^{2}-{\left(\sum_{j=1}^{n}w_{i\,j}\right)}^{2}\right]}{n-1}}}$$

Gi* statistics is an indicator of local aggregation. If the value of Gi* is greater than 0, it indicates that the neighbor attribute value of its spatial unit i is high; otherwise, the neighbor attribute value is low. Local spatial autocorrelation at the county level is reflected by the local indicators of spatial association (LISA). The LISA clustering map involves four patterns: high-high, high-low, low-high, and low-low.

### Spatio-temporal clustering analysis

Spatio-temporal statistics were employed to describe the temporal and spatial distribution, and to detect the geographic and temporal clusters of VL during 2019–2021. A Poisson model using a retrospective space–time permutation scan statistic was used to identify spatio-temporal clusters of VL using SatScan software version 9.4.2 (Satscan). Space–time scanning, defined as a dynamic scan using a cylindrical window in dimensions of time scales and geographical locations, was also used in the identification of spatio-temporal disease clusters. The log-likelihood ratio (LLR) is used as a measure of change in the time and space in a cylindrical window (Kulldorff et al., 1998), and the relative risk (RR) was also calculated. All incidence data were processed separately for each year for which data were available, and potential spatial clusters were detected using a restricted log-likelihood ratio (RLLR). A Monte Carlo simulation was used for permutation testing with statistical significance judged by *p*-value. A *p-value* < 0.05 was indicative of a statistically significant cluster. The grade is based on the value of the log-likelihood ratio (LLR) to generate Grade I, Grade II, Grade III, and Grade IV cluster areas. According to the *P*-value and LLR value, it is determined whether there is clustering in the study area, and its exact location and risk size are also analyzed. Finally, the result was visualized through ESRI ArcGIS software (version 10.3).

## Results

### General status

Between 2019 and 2021, a total of 612 VL cases were reported in China, including one fatality. The annual incidence increased year by year, with 161, 214, and 237 cases reported each year, respectively. After the epidemiological investigation of individual cases, the origin of 608 cases, including 10 cases of AVL, 20 cases of DT-ZVL, and 578 cases of MT-ZVL, can be traced to the county level. However, the other four cases could not be traced to the source of infection and were not included in the epidemiological analysis (Table 1).

**TABLE 1:** Number of visceral leishmaniasis cases reported in China between 2019 and 2021.

Year	Endemic areas			Total	*n*/%
	
	AVL	DT-ZVL	MT-ZVL		
2019	0	8	150	158	25.99
2020	5	4	204	213	35.03
2021	5	8	224	237	38.98
Total	10	20	578	608	100

AVL, anthroponotic visceral leishmaniasis; MT-ZVL, mountain-type zoonotic visceral leishmaniasis; DT-ZVL, desert-type zoonotic visceral leishmaniasis.

### Demographic distribution

The VL cases were mainly distributed in the age group ≥ 15 years old, accounting for 69.57% (423/608) of the total cases. AVL, DT-ZVL, and MT-ZVL cases were mainly distributed in the age group ≥ 15 years old, accounting for 60.00, 65.00, and 69.90% of the total cases of each type, respectively (Table 2).

**TABLE 2:** Demographic distribution of visceral leishmaniasis cases in China between 2019 and 2021.

Characters	Endemic area						Total	
	
	AVL		DT-ZVL		MT-ZVL			
				
	No. of case	*n*/%	No. of case	*n*/%	No. of case	*n*/%	No. of case	*n*/%
**Age group/year**								
0–2	1	10.00	7	35.00	118	20.42	126	20.72
3–6	1	10.00	0	0.00	39	6.75	40	6.57
7–14	2	20.00	0	0.00	17	2.94	19	3.13
≥15	6	60.00	13	65.00	404	69.90	423	69.57
**Occupation**								
Infants and young children	2	20.00	7	35.00	156	26.99	165	27.14
Students	3	30.00	0	0.00	25	4.33	28	4.61
Peasants	4	40.00	10	50.00	271	46.89	285	46.88
Workers	0	0.00	0	0.00	31	5.36	31	5.10
Officials	0	0.00	0	0.00	7	1.21	7	1.15
Housewives	0	0.00	0	0.00	39	6.75	39	6.41
Others	1	10.00	3	15.00	49	8.48	53	8.72
**Gender**								
Male	5	50.00	16	80.00	396	68.51	417	68.59
Female	5	50.00	4	20.00	182	31.49	191	31.41
Total	10	100.0	20	100.0	578	100.0	608	100.0

AVL, anthroponotic visceral leishmaniasis; MT-ZVL, mountain-type zoonotic visceral leishmaniasis; DT-ZVL, desert-type zoonotic visceral leishmaniasis.

In terms of occupation, peasants are the highest risk population for VL (285/608), followed by infants and young children (165/608). For AVL, the predominant cases (4/10) were peasants. For DT-ZVL, most of the cases were peasants (10/20). For MT-ZVL, the majority of cases were also peasants (271/578) followed by infants and young children (156/578; Table 2).

In addition, the male/female ratio of VL cases in China was 421:191, and the male cases were significantly higher than the female cases. The ratio of males to females was 1:1, 1:0.25, and 1:0.46 for AVL, DT-ZVL, and MT-ZVL, respectively (Table 2).

### Temporal distribution

**FIGURE 1:**
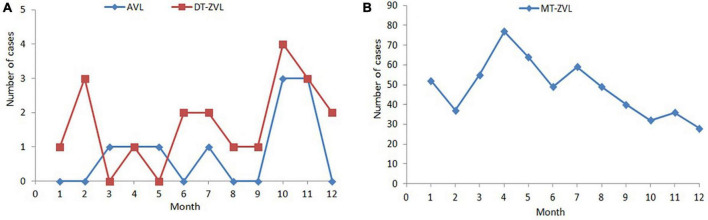
Monthly distribution of visceral leishmaniasis cases in China between 2019 and 2021. **(A)** Monthly distribution of anthroponotic visceral leishmaniasis and desert-type zoonotic visceral leishmaniasis cases. **(B)** Monthly distribution of mountain-type zoonotic visceral leishmaniasis.

The incidence peak varied in different types of VL. For MT-ZVL, it appeared in the period from March to May. However, no obvious peak of AVL and DT-ZVL was observed due to the low number of cases (Figure 1).

### Epidemic trend of visceral leishmaniasis from 2015 to 2021

**FIGURE 2:**
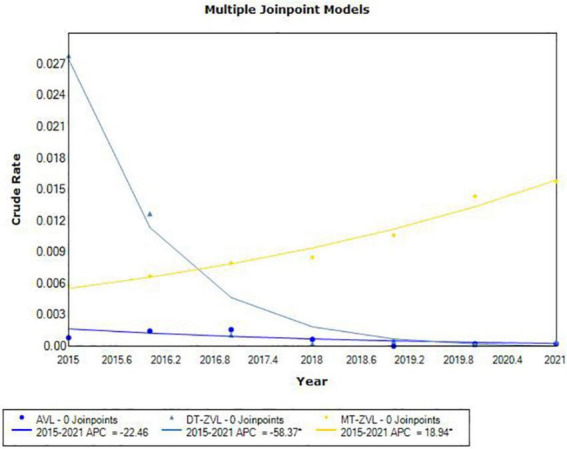
The joinpoint regression analysis for determining changes in the trend of visceral leishmaniasis incidence in China from 2015 to 2021.

JoinPoint regression analysis showed different incidence trends in three types of VL between 2015 and 2021. The incidence of MT-ZVL increased significantly (APC = 18.94, *P* < 0.05). On the contrary, the incidence of DT-ZVL decreased rapidly (APC = -58.37, *P* < 0.05; Figure 2).

### Analysis of spatial-temporal characteristics geographic distribution

**FIGURE 3:**
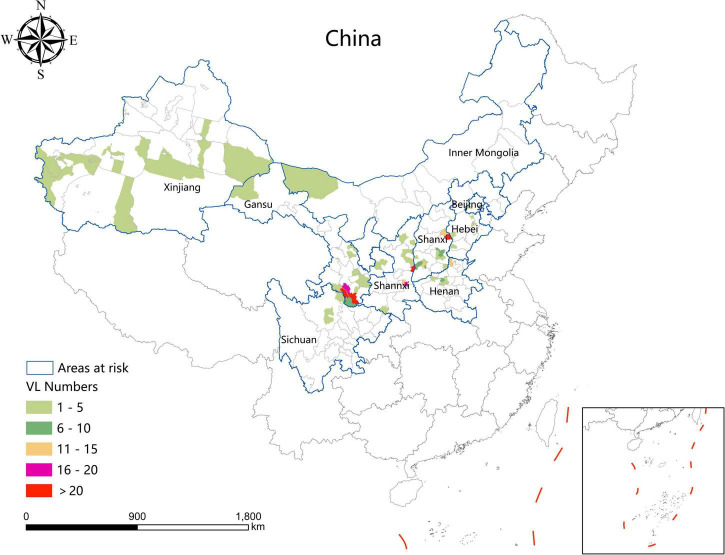
Cumulative number of visceral leishmaniasis cases between 2019 and 2021.

During the study period, the areas with a high incidence of VL were mainly concentrated in southern Gansu, eastern Shaanxi (Huazhou, Linwei District), the Shaanxi–Shanxi border areas (Hancheng city, Hejin City, and Xiangning County), eastern Shanxi (Yangquan City), and northern Henan Province (Linzhou City). In addition, a total of 54 VL cases were detected in 24 resurgence counties which were mainly located in Henan (8), Shanxi (6), Hebei (5), Shaanxi (4), and Gansu (3) Provinces, and Beijing Municipality (1) (Figure 3).

### Spatial autocorrelation analysis

**FIGURE 4:**
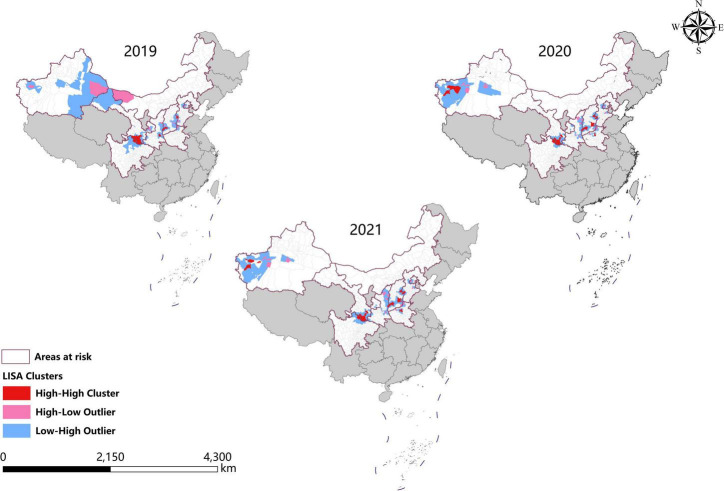
Spatial autocorrelation analysis of visceral leishmaniasis incidence in mainland China from 2019 to 2021.

In the last 3 years, the high-high clusters were mainly observed in the eastern Shanxi (Yangquan), the border areas of Shaanxi–Shanxi (Hancheng, Hejin, and Linfen), southern Gansu and northern Sichuan (Wudu, Zhouqu, and Jiuzhaigou), and southeast Shanxi Province (Changzhi). Since 2020, new high-high clusters began to occur in the northwestern Henan Province (Zhengzhou) and southern Xinjiang (Kashi Prefecture). In 2019, high-low clustering appeared in western Inner Mongolia (Ejina Banner) and eastern Xinjiang (Hami City), since sporadic cases were reported in these sparsely populated areas (Table 3 and Figure 4).

**TABLE 3:** Spatial autocorrelation analysis of visceral leishmaniasis incidence in mainland China from 2019 to 2021.

Year	Moran’s *I*	Variance	*Z* value	*P*-value	Distributed
2019	0.014934	0.000002	11.017085	<0.05	Clustered
2020	0.016131	0.000001	13.618712	<0.05	Clustered
2021	0.017449	0.000002	12.151884	<0.05	Clustered

### Hotspots analysis

**FIGURE 5:**
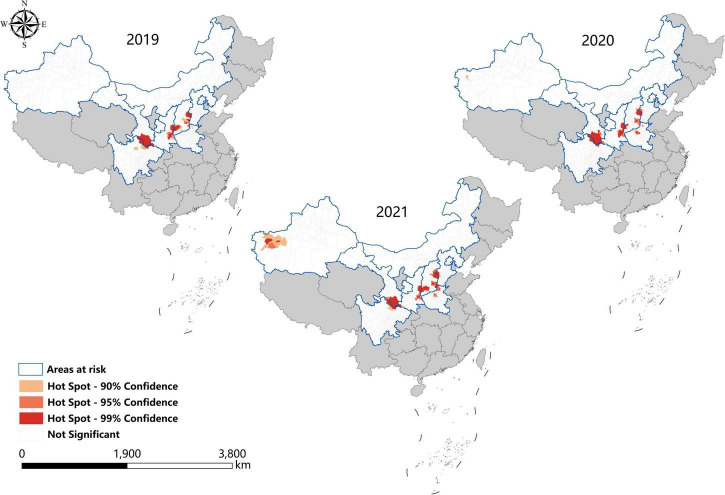
Hotspot analysis of visceral leishmaniasis incidence in mainland China from 2019 to 2021.

Consistent with the result of spatial autocorrelation analysis, hotspots were predominantly identified in eastern Shanxi, the border areas of Shaanxi–Shanxi, southern Gansu, and northern Sichuan, as well as southeast Shanxi Province (Changzhi), from 2019 to 2021. In addition, new hotspots were identified in northwestern Henan Province and southern Xinjiang from 2020 to 2021 (Figure 5).

### Spatio-temporal clustering analysis

**FIGURE 6:**
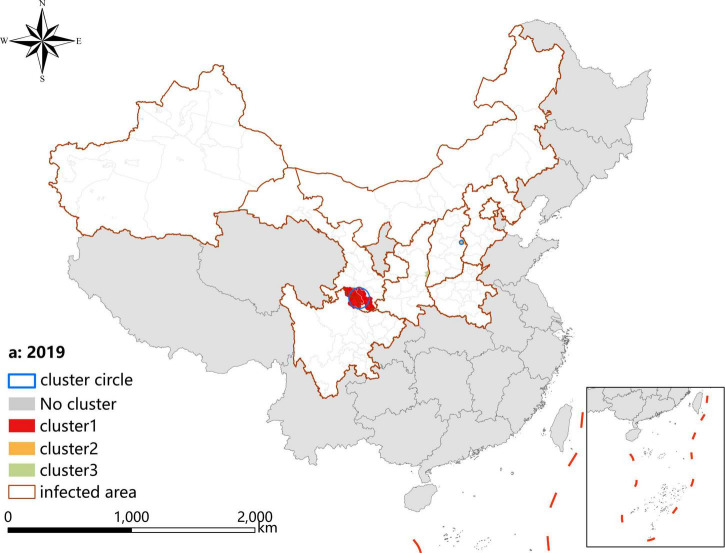
Spatio-temporal clustering analysis of visceral leishmaniasis incidence in mainland China in 2019.

**FIGURE 7:**
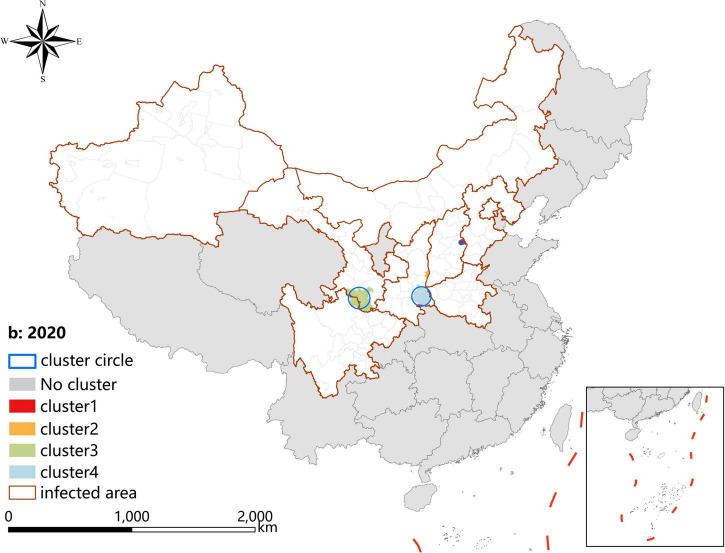
Spatio-temporal clustering analysis of visceral leishmaniasis incidence in mainland China in 2020.

**FIGURE 8:**
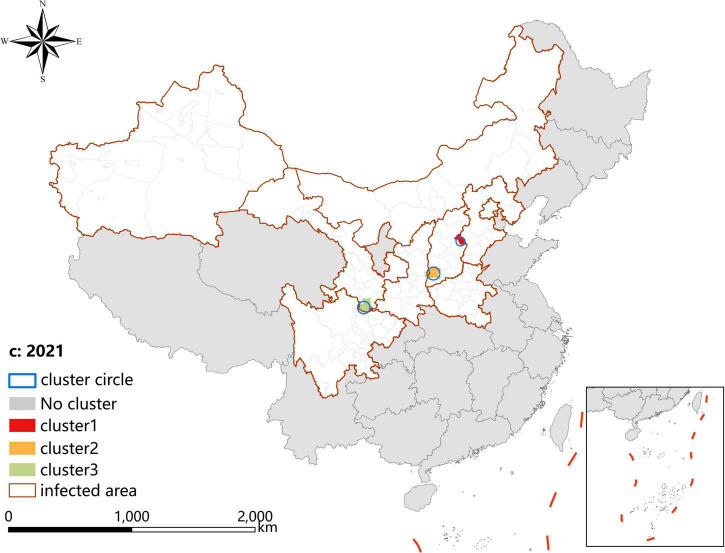
Spatio-temporal clustering analysis of visceral leishmaniasis incidence in mainland China in 2021.

A *p*-value < 0.05 was indicative of a statistically significant cluster.The result, using LLR to estimate the degree of infection clustering (Table 4), showed that 3, 4, and 3 statistically significant spatio-temporal clusters were detected at the county level each year of 2019–2021, respectively. The clusters detected in southern Gansu and northern Sichuan Province degraded from Grade I in 2019 to Grade III in 2020 and 2021. However, the clusters identified in eastern Shanxi Province (Yangquan City) upgraded from Grade II in 2019 to Grade I in 2020 and 2021. In addition, the cluster located in Shaanxi–Shanxi border areas upgraded from Grade III in 2020 to Grade II in 2021 (Figures 6–8).

**TABLE 4:** Spatio-temporal clustering analysis of visceral leishmaniasis incidence in mainland China from 2019 to 2021.

Year	Cluster center		Radius(km)	No. of clustered countries	No. of cases	No. of expected Cases	Relative risk	LLR	*P*-value
	
	Latitude	Longitude							
2019	33.628442	104.319255	83.79	5	31	0.22	174.32	125.48	<0.05
	37.851191	113.591199	14.60	4	24	0.21	137.98	92.30	<0.05
	35.574249	110.381627	0.00	1	16	0.07	234.34	70.46	<0.05
2020	37.851191	113.591199	14.60	4	56	0.28	279.77	250.25	<0.05
	35.574249	110.381627	0.00	1	19	0.10	203.22	81.12	<0.05
	33.628442	104.319255	87.13	7	22	0.49	50.65	63.54	<0.05
	33.891805	109.861779	78.36	10	9	0.80	11.71	13.74	<0.05
2021	37.925958	113.524286	36.34	5	59	0.39	207.11	246.57	<0.05
	35.591589	110.951950	52.16	10	34	0.95	42.16	91.28	<0.05
	32.942840	104.774279	50.31	2	9	0.20	47.31	25.72	<0.05

## Discussion

As a momentous parasitic disease, VL has been brought under control in China (Wang et al., 2000). However, with the development of society and the improvement in the ecological environment, the incidence of MT-ZVL rocketed rapidly and generated new hotspots in central China recently (Zhou et al., 2020a,b; Guan et al., 2021; Hao et al., 2021). Therefore, the objective of this study was to retrospectively analyze the epidemiology and spatiotemporal distribution characteristics of VL in China from 2019 to 2021, aiming to provide insights into the prevention and control of VL.

Between 2019 and 2021, a total of 612 VL cases were reported in endemic areas of China, mostly in Shanxi (48.86%), Shaanxi (19.28%), and Gansu (17.32%), rather than Xinjiang Uygur Autonomous Region which was the predominant former endemic area. It indicates that the main endemic areas shifted from the Xinjiang Uygur Autonomous Region in western China to Shanxi, Shaanxi, and Gansu Province in central China (Han et al., 2019; Zhou et al., 2020c; Guan et al., 2021). During the study period, the cases of AVL, DT-ZVL, and MT-ZVL accounted for 1.63, 3.27, and 94.44% of the total number of reported cases in the country, respectively, indicating that the predominant epidemic type of VL has changed from DT-ZVL to MT-ZVL (Han et al., 2019; Zhou et al., 2020c; Guan et al., 2021). Joinpoint regression analysis showed an upward trend (APC = 18.94, *P* < 0.05) in the incidence of MT-ZVL in China from 2015 to 2021, most notably in Shanxi and Shaanxi Provinces, revealing an observable increase in the incidence of MT-ZVL in these areas. On the contrary, the incidence of DT-ZVL decreased rapidly (APC = -58.37, *P* < 0.05). It may be due to the large-scale renovations of old housing constructions supported by poverty alleviation campaigns in rural areas, particularly in southern Xinjiang which was the most affected area in China. The greatly improved housing conditions of residents destroyed the breeding sites of peridomestic sand flies, and reduced the frequency of contact between the population and sand flies (Zhou et al., 2020c).

Three types of VL exhibit substantially different epidemiological characteristics in China (Wang et al., 2012; Zheng et al., 2017). AVL is mostly endemic in the oasis area of southern Xinjiang, and DT-ZVL is primarily located in the desert area of southern Xinjiang (Zhou et al., 2007; Wang et al., 2012). In this study, the cases of AVL and DT-ZVL were not sufficient to perform a demographic distribution analysis. For MT-ZVL, it mainly occurred in the mountainous regions of western and central China (Zheng et al., 2017; Han et al., 2019; Zhou et al., 2020b), and the principal patients were farmers, followed by infants and young children aged 6 years and below, who constituted high-risk population who need to be targeted with prevention and control measures.

The substantial increase in the incidence of MT-ZVL in China may be related to the emergence of VL in the historically endemic areas and the formation of some new hotspot areas of MT-ZVL (Zhou et al., 2020c; Guan et al., 2021; Hao et al., 2021). Between 2019 and 2021, a total of 27 new reemerged historically endemic counties were identified that were mainly located in Shanxi, Shaanxi, Henan, and Hebei Provinces and Beijing municipality, where 54 VL cases were notified. Several factors contributed to the reemergence of MT-ZVL. First, the number of roaming dogs increased the spread of VL from the natural nidus, where the circulation of VL was maintained in the wild animals, to the residential areas. Second, imported dogs with leishmaniasis facilitated the reestablishment of VL, while sandflies widely infested mountainous areas (Zhou et al., 2020c).

Spatial analysis showed highly consistent hotspots and clustering regions by different methods (spatial autocorrelation and hotspot analysis), particularly for the high-risk region (Grade I), revealing spatial clustering of VL in the whole country for each year of the study period. In the past 3 years, the high-incidence clusters were maintained mainly in the eastern and southeast Shanxi Province, the border of Shanxi Province, and Shaanxi Province, as well as the border of southern Gansu and northern Sichuan regions, demonstrating that although southern Gansu and northern Sichuan were still traditionally the high-incidence regions, two new higher-incidence regions have formed in Yangquan City and Hancheng City in recent years, where they have become the most predominant endemic areas of VL in China. The spatio-temporal cluster analysis also showed similar results that the clusters located in eastern Shanxi Province (Yangquan City) and Shaanxi–Shanxi border areas have surpassed those located in southern Gansu and northern Sichuan, and became the top grade clusters. These areas are extension regions of the Loess Plateau and are mainly hilly settings, where the Yanshan-Taihangshan mountain deciduous broad-leaved forest ecological zone and Fenwei Basin Agro-ecological zone are located (Gong et al., 2021). The hilly and frondent environments provide a favorable habitat for a large number of wild animals and sandflies, making it a suitable natural endemic focus of MT-ZVL. Since the 1990s, with the rapid development of urbanization in China, the rural population has decreased dramatically, and the vigorous promotion of ecological protection measures in China, such as returning farmland to forests, has provided a suitable ecological environment in the countryside of these areas for the reproduction of *Phlebotomus sinensis*(*Ph. sinensis*), to the extent that the density of *Ph. sinensis* in Yangquan City has reached as high as 103 sandflies/per person per hour recently (Luo et al., 2022). At the same time, a huge number of dogs are kept in these areas as watchdogs, sheeplings, and pets, abundant of which are stray dogs with infection rates of canine visceral leishmaniasis as high as 30% (Li et al., 2021a,b). In comparison with the region of southern Gansu and northern Sichuan, these areas possess a better breeding condition for *Ph. sinensis*, such as lower elevation, higher temperature, longer active season, and more moist and loose soil, so they have higher vector capacity for the transmission of VL. Given all this, it is no surprise that Yangquan City and Hancheng City developed into the highest incidence regions of VL in China.

Since 2020, small clusters located in southern Xinjiang indicated that risks of DT-ZVL still existed in the desert areas of southern Xinjiang, so we still need to pay close attention to these areas in case of their cyclical outbreaks. Scattered small clusters that appeared in northwestern Henan, southeast Shanxi, western Hebei, and northern Beijing showed that MT-ZVL is reemerging in the Yanshan-Taihangshan mountain regions. Therefore, there is an urgent need for better cooperation among multi-sectors of government for the prevention and control of further extension of MT-ZVL in the surrounding regions.

Several phenomena discovered recently have made the prevention and control of VL more complicated. The first is that autochthonic cases, canine leishmaniasis, and *Ph. sinensis* have been detected in the densely populated main city of Yangquan, indicating that there is a trend of urbanization of MT-ZVL, which has been reported in Europe and South America (Dujardin, 2006; World Health Organization [WHO], 2002). The second is that MT-ZVL is spreading from lower latitudes to higher latitude areas in China due to global warming, and this trend has been seen in Germany and Italy (Ferroglio et al., 2005; Naucke et al., 2008), indicating further expansion of the suitable area for VL transmission. The third is that a large amount of reemerged historically endemic counties have been detected in the mountain areas of central and Western China, indicating that the ecological and geographical environment is favorable for the resurgence and even the outbreak of MT-ZVL. Therefore, control efforts should be strengthened to prevent further growth of MT-ZVL cases. Prevention of infection can be achieved by utilizing insecticide-impregnated collars, the effectiveness of which directly depends on the coverage and loss rate. Stray dogs may function as infection reservoirs if not targeted, so stray dog population management should be an important part of VL control programs (Zhou et al., 2020c). Strictly, MSZ-VL is a zoonotic disease with both dogs and wild animals serving as reservoir hosts, so it is a big challenge to completely eliminate the disease. The government must establish a collaboration mechanism with multisectors involved, such as the Centers for Disease Control and Prevention, hospitals, and Veterinary Department and Public Security Department. It is also necessary to provide long-term necessary policy and financial support for routine surveillance and control programs.

## Conclusion

This study showed that the number of DT-ZVL and AVL cases decreased, while MT-ZVL cases increased quickly. Therefore, it is urgent to strengthen organizational leadership and systematically carry out necessary interventions to prevent further growth of MT-ZVL cases in hotspots and clustering areas, especially in Shanxi and Shaanxi Provinces.

## Data availability statement

The raw data supporting the conclusions of this article will be made available by the authors, without undue reservation.

## Author contributions

YL, ZZ, and SL: conceptualization. YL, ZWL, YH, ZZ, and SL: data curation. YL, ZWL, ZZ, and SL: formal analysis and methodology. YL, ZWL, YH, YZ, ZZ, and SL: funding acquisition. YL, LY, ZQL, and ZZ: investigation. YZ and SL: project administration and supervision. ZWL: software. YL, ZWL, and ZZ: writing of the original draft. ZZ, YZ, and SL: reviewing and editing. All authors contributed to the article and approved the submitted version.
